# The Potential Role of sPD-L1 as a Predictive Biomarker in EGFR-Positive Non-Small-Cell Lung Cancer

**DOI:** 10.3390/cimb47010045

**Published:** 2025-01-11

**Authors:** Vesna Ćeriman Krstić, Dragana Jovanović, Natalija Samardžić, Milija Gajić, Jelena Kotur Stevuljević, Aleksandra Klisic, Ivan Soldatović, Damir Radončić, Marina Roksandić Milenković, Biljana Šeha, Nikola Čolić, Katarina Lukić, Milan Savić

**Affiliations:** 1 Faculty of Medicine, University of Belgrade, 11000 Belgrade, Serbia; drcola12@gmail.com (N.Č.); drmilansavic@gmail.com (M.S.); 2 Clinic for Pulmonology, University Clinical Center of Serbia, 11000 Belgrade, Serbia; natalis.dm@gmail.com (N.S.); milijagajic@gmail.com (M.G.); damirradoncic@hotmail.com (D.R.); 3 Internal Medicine Clinic “Akta Medica”, 11000 Belgrade, Serbia; draganajv@yahoo.com; 4 Department for Medical Biochemistry, Faculty of Pharmacy, University of Belgrade, 11000 Belgrade, Serbia; jelena.kotur@pharmacy.bg.ac.rs; 5 Faculty of Medicine, University of Montenegro, 81000 Podgorica, Montenegro; aleksandranklisic@gmail.com; 6 Center for Laboratory Diagnostics, Primary Health Care Center, 81000 Podgorica, Montenegro; 7 Institute of Medical Statistics, Faculty of Medicine, University of Belgrade, 11000 Belgrade, Serbia; soldatovic.ivan@gmail.com; 8 Municipal Institute for Lung Diseases and TB, 11000 Belgrade, Serbia; dr.marr@gmail.com; 9 Clinic for Neurosurgery, University Clinical Center of Serbia, 11000 Belgrade, Serbia; biljana.seha@yahoo.com; 10 Center for Radiology, University Clinical Center of Serbia, 11000 Belgrade, Serbia; katarina.krstic86@hotmail.com; 11 Clinic for Thoracic Surgery, University Clinical Center of Serbia, 11000 Belgrade, Serbia

**Keywords:** NSCLC, EGFR, sPD-L1, PD-L1, immunotherapy, molecular therapy, biomarker

## Abstract

Background/Objectives: A significant breakthrough in non-small-cell lung cancer (NSCLC) treatment has occurred with the introduction of targeted therapies and immunotherapy. However, not all patients treated with these therapies would respond to treatment, and patients who respond to treatment would acquire resistance at some time point. This is why we need new biomarkers that can predict response to therapy. The aim of this study was to investigate whether soluble programmed cell death-ligand 1 (sPD-L1) could be a predictive biomarker in patients with epidermal growth factor receptor (EGFR)-positive NSCLC. Materials and Methods: Blood samples from 35 patients with EGFR-mutated (EGFRmut) adenocarcinoma who achieved disease control with EGFR tyrosine kinase inhibitor (EGFR TKI) therapy were collected for sPD-L1 analysis. We analyzed sPD-L1 concentrations in 30 healthy middle-aged subjects, as a control population, to determine the reference range. Adenocarcinoma patients were divided into two groups, i.e., a group with low sPD-L1 (≤182.5 ng/L) and a group with high sPD-L1 (>182.5 ng/L). Results: We found that progression-free survival (PFS) was 18 months, 95% CI (11.1–24.9), for patients with low sPD-L1 and 25 months, 95% CI (8.3–41.7), for patients with high sPD-L1. There was no statistically significant difference in PFS between the groups (*p* = 0.100). Overall survival (OS) was 34.4 months, 95% CI (26.6–42.2), for patients with low sPD-L1 and 84.1 months, 95% CI (50.6–117.6), for patients with high sPD-L1; there was also no statistically significant difference between the groups (*p* = 0.114). Conclusion: In our study, we found that patients with high sPD-L1 had numerically better PFS and OS, but this has no statistical significance. Further studies with a larger number of patients are needed to evaluate the role of sPD-L1 as a predictive biomarker in patients with EGFRmut NSCLC.

## 1. Introduction

Lung cancer remains a leading cause of cancer-related deaths worldwide [1,2]. Despite significant advances in treatment, non-small-cell lung cancer (NSCLC) remains an incurable disease for the majority of patients [1]. For a long time, chemotherapy was the standard of care for patients with the advanced stage of the disease. For these patients, the five-year overall survival (OS) was less than 5% [2]. A big step was made with the introduction of targeted therapies and immunotherapy.

The activation of the PD-1/PD-L1 pathway leads to the exhaustion of T-cells and continuous tumor growth [1]. Programmed cell death protein 1 (PD-1)/programmed cell death-ligand 1 (PD-L1) antibodies have demonstrated impressive results in the treatment of patients with metastatic NSCLC without targetable mutations [1]. However, a significant proportion of patients experienced disease progression shortly after starting immunotherapy even after biomarker selection [3], such as PD-L1. Some studies have shown that the upregulation of the PD-1/PD-L1 pathway was associated with resistance to epidermal growth factor receptor (EGFR)–tyrosine kinase inhibitor (TKI) therapy [1,4,5,6]. It was also reported that high levels of membrane PD-L1 were correlated with primary resistance and a poor response to EGFR TKIs [1,4,5,6]. One of them found that PD-L1 expression was increased in patients who acquired resistance to EGFR TKIs [4], whereas several others have reported a correlation between high PD-L1 expression and primary resistance and poor response to EGFR TKIs [5,6].

It was found that PD-L1 exists as a membrane-bound, soluble form [7] and in exosomes [8]. No correlation was found between membrane PD-L1 and the soluble form, and it has been acknowledged that the tumor microenvironment and nonmalignant cells may generate soluble programmed cell death-ligand 1 (sPD-L1) as well [9]. It could be found on the surface of the various cells, and in addition, sPD-L1 can be released from PD-L1-positive tumor cells or immune cells [10]. sPD-L1 can be detected not only in patients with carcinomas, but also in other diseases and in the healthy population with the highest levels in adults between 51 and 70 years [7,9]. It can also be found in the plasma and in other liquids such as pleural effusion in lung cancer patients, but also in the supernatant of mPD-L1-positive malignant cell lines, and it is a product of mPD-L1 cleavage [7]. Also, it was shown that besides the proteolytic process of mPD-L1/exoPL-L1, sPD-L1 can arise from the translation of alternatively spliced PD-L1 mRNA. Different variants of sPD-L1 can arise, which are structurally and functionally variable. It was speculated that two variants, PD-L1v1 and PD-L1v4 mRNA, were more expressed in cancer cells than in normal tissue [11,12].

Sagawa et al. [13] in their research found that the secretion of PD-L1v4 resisted anti-PD-L1 antibody therapy compared to wild-type PD-L1, which was explained as a decoy effect. They concluded that the decoy effect of PD-L1 splicing variants may be one of the mechanisms of treatment resistance.

However, it has been proven that sPD-L1 holds its biological activity, and it is able to specifically bind to the PD-1 receptor in peripheral blood, thus activating the PD-1/PD-L1 pathway [10]. It could be the way how sPD-L1 achieves a systematic immunosuppressive effect.

The exosomes are membrane-bound vesicles, which can be found in blood, urine, and other body fluids [8]. They are secreted by tumor cells under oxidative stress [8]. It was confirmed that tumor cells can release exosomes carrying PD-1, PD-L1, and cytotoxic T-lymphocyte-associated antigen 4 (CTLA-4) [8].

It was found that EGFR mutations were directly associated with PD-L1 upregulation in NSCLC patients, which could lead to increased risk of tumor immune escape [1,14]. There is evidence that activated STAT3 (signaling transducer and activator of transcription 3) binds to the PD-L1 promoter and, in that way, promotes the transcription of PD-L1 [1,14]. It was shown that the EGFR signaling pathway was involved in the activation of the IL-6/Janus kinase (JAK)/STAT3 pathway [1,14,15]. This could imply that EGFR signaling could regulate PD-L1 expression via the IL-6/JAK/STAT3 pathway. EGFR activation has been found to upregulate PD-L1 through p-ERK1/2/p-c-Jun [16].

There is large heterogeneity among studies that have explored the role of soluble PD-L1 (sPD-L1) as a prognostic and/or predictive biomarker [8,10,17]. Different tests have been used to detect sPD-L1, but there is no consensus on the range of positive results.

The purpose of this pilot study is to investigate whether sPD-L1 could potentially be a predictive biomarker in patients with EGFR-mutated (EGFRmut) lung adenocarcinoma treated with EGFR TKIs based on a comparison of sPD-L1 blood levels and clinical outcomes. Since the samples are obtained from peripheral blood, it is suggested that it might be a valuable source of material for monitoring responses to therapy and further treatment decisions, as liquid biopsy is non-invasive, easy to perform, and can be repeated at any time point.

## 2. Materials and Methods

### 2.1. Patients and Data Collection

Patients with EGFRmut advanced lung adenocarcinoma who were treated at the Clinic for Pulmonology, University Clinical Center of Serbia, from February 2012 until January 2017 were enrolled in this study. All patients were treated with first-line EGFR TKIs: gefitinib, erlotinib, and afatinib. Data on sex, age, smoking status [i.e., non-smokers, ex-smokers (patients who stopped smoking one year before treatment), and smokers], stage of disease, response to therapy (ORR), time to disease progression (PFS), and overall survival (OS) were collected. Response to therapy included complete response, partial response, stable disease, and progression of disease.

A responder is defined as a patient who had a complete response (CR) or partial response (PR) for at least 4 weeks during treatment (confirmed response). Disease control is defined as response—as defined above—or stable disease (SD) for at least 6 weeks. PFS is defined as the time from the start of the therapy until disease progression or death, whichever occurs first. OS is defined as the time from the start of the therapy until death.

The source material used in this study was peripheral blood because liquid biopsy is non-invasive, easy to perform, and can be repeated during the treatment. Blood samples were collected from 35 of the patients for sPD-L1 analysis, who achieved disease control with EGFR TKI therapy (these patients had started EGFR TKIs and achieved disease control) and who were alive at the moment the analysis was performed. We also analyzed sPD-L1 concentrations in 30 healthy middle-aged subjects to determine reference ranges for sPD-L1 in the healthy population. Reference limits for the healthy middle-aged population have been calculated by a nonparametric method as 2.5th and 97.5th percentile values [18]. This is the usual method for reference boundary determination for parameters with non-normal distribution, such as PD-L1 [15].

### 2.2. Sample Collection

Blood samples were collected into lithium heparin tubes (BD Diagnostics, Wokingham, UK). Samples were diluted 1:2, and the total volume of the samples used was 100 μL (0.1 mL) as per the manufacturer’s protocol. Plasma was isolated by centrifugation at 1000× *g* RCF for 15 min and stored at −80 °C until analysis. We used the DuoSet ELISA system (R&D Systems Europe, Ltd., Abingdon, UK) for PD-L1 (B7-H1/CD274) determination in plasma as a sandwich enzyme-linked immunosorbent assay (ELISA) specific to the human B7-H1T.

### 2.3. Statistical Analysis

The results are presented as counts (%), means ± standard deviation, or median (25th–75th percentile) depending on data type and distribution. Groups were compared using the nonparametric Pearson Chi-square test. Kaplan–Meier curves with a log-rank tests were used to assess survival and group differences regarding survival. All *p*-values less than 0.05 were considered significant. All data were analyzed using SPSS 29.0 (IBM Corp. Released 2023. IBM SPSS Statistics for Windows, Version 20.0. Armonk, NY, USA: IBM Corp.) and R 3.4.2. (R Core Team (2017). R: A language and environment for statistical computing. R Foundation for Statistical Computing, Vienna, Austria. URL https://www.R-project.org/, accessed on 2 December 2024).

## 3. Results

A total of 105 EGFRmut advanced adenocarcinoma patients were enrolled in this study. Blood samples were collected from 35 of them for sPD-L1 analysis, who achieved disease control and who were still alive at the time the blood was collected.

Reference limits for the healthy middle-aged population were calculated by a nonparametric method as 2.5th and 97.5th percentile values^15^: sPD-L1: 31.0–182.5 ng/L.

The mean sPD-L1 value for the EGFRmut adenocarcinoma group of patients was 155.74 ng/L, and 28.6% of the patients had sPD-L1 levels above the upper limit for healthy controls.

Therefore, adenocarcinoma patients were divided into two groups depending on the sPD-L1 level, i.e., a group with low sPD-L1 (≤182.5 ng/L) and a group with high sPD-L1 (>182.5 ng/L).

The main demographic and baseline characteristics of these two groups of patients are presented in Table 1. There are no statistically significant differences between the groups (Chi-square test).

All patients were treated with first-line EGFR TKIs (Gefitinib, Erlotinib, or Afatinib; 33, 1, 1, respectively).

Disease control was achieved in all patients in both groups. A total of 48% of patients in the group with low sPD-L1 and 30% in the group with high sPD-L1 were responders. There were no statistically significant differences between the groups regarding their response to EGFR TKIs (Chi-square test, *p* = 0.206) (Table 2).

**Figure 1:**
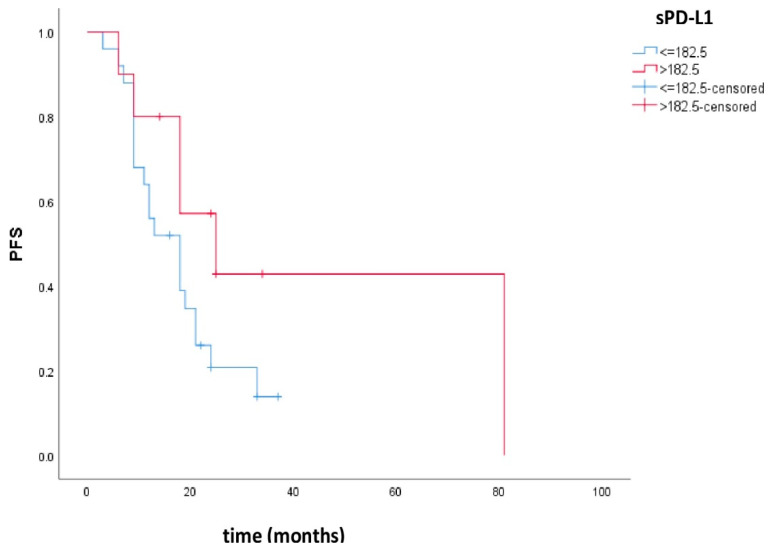
Kaplan–Meier curves of PFS in patients with low and high sPD-L1. Patients with high sPD-L1 levels had better PFS compared to patients with low sPD-L1, but there was no statistical significance (*p* = 0.100).

Progression-free survival was 18 months, 95% CI (11.1–24.9), for patients with low sPD-L1 and 25 months, 95% CI (8.3–41.7), for patients with high sPD-L1. There was no statistically significant difference in PFS between these groups (*p* = 0.100). Figure 1 shows the Kaplan–Meier curves of PFS in patients with low and high sPD-L1.

**Figure 2:**
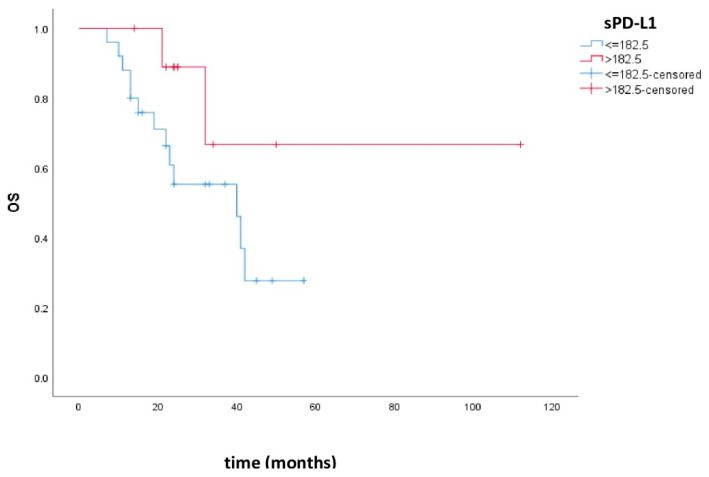
Kaplan–Meier curves of OS in patients with low and high sPD-L1. Patients with high sPD-L1 levels had better OS compared to patients with low sPD-L1, but there was no statistical significance (*p* = 0.114).

Overall survival was 34.4 months, 95% CI (26.6–42.2), in the group of patients with low sPD-L1 and 84.1 months, 95% CI (50.6–117.6), in the group with high sPD-L1. There was no statistically significant difference in OS between these groups (*p* = 0.114). Figure 2 shows the Kaplan–Meier curves of OS in patients with low and high sPD-L1.

## 4. Discussion

The majority of lung cancer studies have shown that patients with elevated sPD-L1 had worse treatment and overall survival outcomes. We conducted this study in patients with EGFRmut lung adenocarcinoma who achieved disease control in order to investigate whether this finding also applies to this group of patients. Considering that there is no consensus on the reference range for sPD-L1, we have also investigated which group of patients in other studies had elevated values.

The mean sPD-L1 value for our group of patients was 155.74 ng/L, and 28.6% of patients had sPD-L1 levels above the upper limit for healthy controls. A possible explanation could be the fact that the majority of our patients achieved disease control, and another reason could be the small number of patients.

In a study by Li et al. [8], there was no significant difference between levels of sPD-L1 in 48 patients with NSCLC and healthy controls. This might be due to the fact that the majority of patients (i.e., 76.5% of them) were at stage I-IIIa and almost 86% did not have distant metastases [8]. They also found that patients with stage II/III/IV had significantly higher levels of sPD-L1 than patients with stage I, but there were no significant differences between the groups [8].

However, in two other studies, it was reported that levels of sPD-L1 were higher in patients with lung cancer than in healthy controls [2,3,17]. However, these studies included patients with advanced NSCLC [2,3,17], and in the previously mentioned study, the majority of patients were at stage I-IIIa [8].

The PFS in our study was 18 months, 95% CI (11.1–24.9), for patients with low sPD-L1 and 25 months, 95% CI (8.3–41.7), for patients with high sPD-L1. There was no statistically significant difference in the PFS between these groups (*p* = 0.100). This could be due to the small number of patients.

The OS was 34.4 months, 95% CI (26.6–42.2), for patients with low sPD-L1 and 84.1 months, 95% CI (50.6–117.6), for patients with high sPD-L1, but there was no statistical significance between the groups (*p* = 0.114). This could also be due to the small number of patients.

A possible explanation for these results, since all our patients achieved disease control, could be the reactivation of T-cells, which led to the initiation of immune response, thus killing the tumor cells and leading to the consequent release of sPD-L1. Another possible explanation for our results could be the presence of co-mutations. As already known, the most common co-mutation in EGFR-mutated NSCLC is tumor protein p53 (TP53). It has been proven that patients with EGFRmut NSCLC who also have TP53-positive mutation have worse prognosis than EGFRmut patients who have TP53 wild-type tumors. Besides this co-mutation, the other most frequent co-mutations include retinoblastoma 1 (RB1), CTNNB1, and phosphoinositide 3-kinases (PIK3)CA. PIK3CA and CTNNB1 are more frequent in patients with advanced stages of disease, while TP53 and RB1 are equally found in patients with early and advanced stages of disease [19].

Jiao et al. [20] conducted a study that investigated the correlation between TP53 and EGFR in patients with metastatic NSCLC. They found that the presence of TP53 was a negative prognostic factor for OS. The rate of TP53 was higher in the EGFRmut group of patients than in the EGFR wild-type (EGFRwt) group [20]. Patients with EGFRwt NSCLC and TP53 wild-type had the best prognosis. The same results were shown in patients with exon 19/21 and the TP53 wild-type [20].

As mentioned previously, most of the studies have shown that patients with high sPD-L1 have worse treatment outcomes, regardless of the type of therapy applied.

The results of a meta-analysis conducted by Wei et al. have shown that patients with solid cancers and high levels of sPD-L1 had a shorter OS than those with low levels of sPD-L1 [10].

Ding et al. [21] conducted a meta-analysis, and they also concluded that higher levels of sPD-L1 were associated with worse OS. Statistical significance was also observed in subgroup analysis stratified by cancer type (hematological neoplasms or non-hematological neoplasms), sample size (more or less than 100), cut-off value of sPD-L1 (more or less than 6.51 ng/mL), and ethnicity (Asian or European) [21].

Jin et al. [22] investigated the role of sPD-L1 in patients with small-cell lung cancer (SCLC). They found significantly higher levels of sPD-L1 in SCLC patients than in healthy controls. Also, higher levels of sPD-L1 have been found in patients who had no response to chemotherapy and in patients who died within the 12 months of the follow-up period [22].

In a study conducted by Jovanovic et al. [23], 47% of EGFRmut adenocarcinoma patients had high sPD-L1, i.e., all patients who were PD-L1-positive (these patients had advanced disease), 43% of patients with EGFRwt NSCLC, 64% of patients with squamous cell carcinoma, and 31% of patients with SCLC. Patients with higher sPD-L1 levels had shorter overall survival compared with patients with lower sPD-L1 levels, but without statistical significance. Also, higher sPD-L1 levels have been observed in patients with more advanced stages of disease [23].

There were 15 patients with EGFRmut adenocarcinoma who were included in this study [23]. These patients had lower baseline sPD-L1 levels than patients with EGFRwt NSCLC: 134.4 ng/L vs. 161.4 ng/L, respectively [23]. This difference was not statistically significant, and in our study, the mean sPD-L1 level was 155.74 ng/L. Our result was closer to that of the EGFRwt group of patients. A possible reason for such discrepancies could be the fact that we collected samples from patients who achieved disease control, whereas in the study by Jovanovic [23], the samples were collected at baseline.

Okuma et al. [2] found results similar to those of the majority of published papers. Patients with advanced lung cancer and high sPD-L1 had worse prognoses compared to patients with low sPD-L1 levels [2,3]. No correlation was noted among sPD-L1 levels and age, sex, Eastern Cooperative Oncology Group Performance Status (ECOG PS), histological subtypes, mutational status, stage of disease, smoking status, and previous history of radiotherapy [2,3]. In this study, there were 19 EGFRmut patients and 3 patients with ALK rearrangements [2]. The authors found higher levels of sPD-L1 in these patients than in EGFRwt- and ALK-negative patients, but this difference was not statistically significant [2]. They did not investigate the correlation between the outcomes of EGFR TKI-treated patients and levels of sPD-L1. However, this study was conducted before the immunotherapy had been introduced; therefore, those patients were treated with chemotherapy. Most of the patients were chemotherapy-naïve (67.7%), and the levels of sPD-L1 were similar with the levels of sPD-L1 in the group of previously treated patients [2].

Jia et al. [1] investigated whether sPD-L1 could predict responses to EGFR TKIs in patients with EGFRmut lung adenocarcinoma. They found that patients with high pre-treatment or on-treatment sPD-L1 levels had decreased ORR compared with patients with low sPD-L1 [1]. There were no differences in the treatment response between patients with or without a reduction in sPD-L1 levels [1]. They also found that patients with high baseline sPD-L1 levels and that patients with high on-treatment sPD-L1 levels had shortened PFS, but this difference was not statistically significant [1]. The median PFS for the whole group was 12.5 months, but patients with lower pre-treatment levels of sPD-L1 had statistically superior PFS (16.1 months) compared to patients with higher pre-treatment sPD-L1 levels (9.9 months) [1]. Patients with higher on-treatment levels of sPD-L1 also had shorter PFS, but there was no statistical significance [1]. They did not investigate the association between sPD-L1 and overall survival. No association was observed between sPD-L1 levels and T790M status at the time of progression [1]. Other clinical characteristics such as gender, age, ECOG PS score, tumor stage, smoking status, EGFR status, and type of EGFR TKI received were not associated with the therapeutic response [1].

Zhang et al. [24] conducted a study that included 72 patients with EGFRmut NSCLC and also 31 patients with EGFR wild-type NSCLC as a control group. They found lower levels of sPD-L1 in the EGFRmut group of patients compared to the control group [7,24]. Patients with EGFRmut adenocarcinoma were divided into two groups depending on response after two months of EGFR TKI therapy: the disease progression group and the disease control group. The results showed lower levels of sPD-L1 after EGFR TKIs and significantly higher sPD-L1 levels in the disease progression group compared to the disease control group [7,24].

In another study, Zhang et al. [17] found that patients with low levels of sPD-L1 had longer OS. The same results were found in the EGFRmut group of patients (24 of 73 patients), but there was no statistical significance [3,9,17]. This study was also conducted before the immunotherapy era.

Zhao et al. [25] investigated the levels of sPD-L1 in patients treated with radiotherapy (RT) or concurrent chemotherapy and radiotherapy. They reported that lower levels of baseline sPD-L1 were correlated with longer OS in NSCLC patients treated with RT only [3,25]. Also, sPD-L1 levels were significantly lower at week 2 and week 4 when compared to baseline before RT, but not after RT [3,25]. sPD-L1 levels recovered to the baseline after RT, which may be explained by an increase in inflammation [25]. In the group of patients treated with concurrent chemotherapy and RT, OS was not significantly different between those with low and high baseline sPD-L1 [25]. A subgroup of patients treated with a high dose of RT and having low baseline sPD-L1 levels had the longest survival [25].

Levels of sPD-L1 were also investigated in patients treated with immunotherapy.

Okuma et al. [26] included 39 patients with NSCLC who were treated with nivolumab in a second-line setting or beyond. Plasma samples were collected at baseline. Patients with high sPD-L1 levels had shorter OS and shorter PFS compared to patients with low sPD-L1 levels [26]. The ORR was higher in the group with low sPD-L1 levels compared to the group with high sPD-L1 levels [26].

Another study conducted by Costantini et al. [27] included 43 patients with advanced NSCLC (EGFRwt- and ALK-negative) treated with nivolumab in their second-line treatment or beyond. Samples were collected upon initial diagnosis, before the initiation of nivolumab and again after two months of therapy. There was no difference in sPD-L1 levels collected upon the initial diagnosis and before the initiation of nivolumab between responders and non-responders to nivolumab [27]. However, after two months of nivolumab, sPD-L1 levels were significantly higher in non-responders [27]. An increase in sPD-L1 levels after two months of treatment was associated with lower overall response rates compared to patients who had decreased or stable sPD-L1 levels [27]. They also found that high sPD-L1 levels were correlated with shorter PFS and OS [27].

Bonomi et al. [28] conducted a study with 20 patients with NSCLC and ECOG PS 2. Patients were divided into two groups. One group of patients was treated with pembrolizumab monotherapy, and the other group of patients was treated with combination therapy: pembrolizumab plus carboplatin/paclitaxel. Blood samples were collected at baseline, at week 4, and at week 7. They found that patients with disease progression had higher baseline levels of sPD-L1 compared with patients who had a response to therapy, but this was not statistically significant [28]. No significant changes in levels of sPD-L1 were observed [28].

Dronca et al. [29] reported on 60 NSCLC and melanoma patients who were treated with anti-PD-1 therapy. They found that patients with high baseline sPD-L1 levels had no response to therapy, and they also found increased levels of sPD-L1 after the first tumor assessment in patients who did not have a response to therapy [29].

Wang and He [30] conducted a meta-analysis to identify the prognostic and clinicopathological significance of sPD-L1 in patients with NSCLC. Eleven studies were included in the analysis. They found that high levels of sPD-L1 were correlated with worse OS and PFS [30]. Correlation was not found between levels of sPD-L1 and age, sex, smoking status, ECOG PS, or EGFR mutation status [30].

Zhu and Song [31] found in their research that patients with lung cancer who were treatment-naïve had significantly higher sPD-L1 levels compared to healthy individuals and patients with benign tumors. They also found a positive correlation between poor ECOG PS and the later stage of the disease [31]. sPD-L1 levels were higher in EGFRmut patients compared to EGFRwt patients [31]. This difference was not statistically significant, but there were a small number of patients included in the analysis: 18 EGFRmut and 12 EGFRwt [31]. Also, a correlation was not found between PFS and sPD-L1 levels [31].

Although most of these studies showed worse prognosis or treatment resistance in patients with increased sPD-L1 levels, there are a few studies with results similar to ours. A study conducted by Tiako Meyo et al. [32] investigated the predictive value of biomarkers in advanced NSCLC, including sPD-L1. They included 87 patients, who were divided into two groups: patients treated with nivolumab (51 of them) and a control group of patients with EGFRmut NSCLC (36 of them). Patients treated with nivolumab were older, and 96% of them were former or active smokers, while patients in the EGFRmut group were non-smokers in almost 70% of cases [32]. In the nivolumab group, 52.9% of the patients had a positive sPD-L1 level, while all the patients in the EGFRmut group had a positive sPD-L1 level. The mean sPD-L1 level was higher in the EGFRmut group [32]. Positive levels of sPD-L1 in the nivolumab group were correlated with shorter PFS [32]. However, such correlation was not found in the EGFRmut group of patients [32]. Interestingly, after two cycles of nivolumab, patients with increased or stable levels of sPD-1 had longer PFS and OS [32]. The reduction in levels of sPD-L1 after two cycles of nivolumab was associated with worse outcomes in a univariate analysis, but these results have not been confirmed in a multivariate analysis [32].

Zheng et al. [33] found that patients with advanced gastric cancer and higher levels of sPD-L1 had better prognoses, similarly to our study. Vecchiarelli et al. [34] investigated whether sPD-L1 testing is feasible, whether its levels are changed after therapy, and also whether the baseline levels of sPD-L1 are correlated with the outcomes of treatment. They included 56 patients with advanced NSCLC, and there were 11 patients with EGFRmut NSCLC who were treated with EGFR TKIs. Only three patients were treated with immunotherapy. They observed that patients tretaed with cehmotherapy had a significant increase in median sPD-L1 after 3 months of treatment. But patients tretaed with TKIs and immunotherapy did not have significant changes in median sPD-L1 levels [34]. No significant differences in ORR, PFS, and OS were observed between patients with high and low sPD-L1 levels [34]. These differences were found neither in the group of patients treated with chemotherapy nor in the group of patients treated with TKIs and immunotherapy [34].

Sorensen et al. [35] showed in 38 EGFRmut NSCLC patients treated with erlotinib that the levels of sPD-1 were higher at the time of disease progression than at baseline. Interestingly, they also found that patients with an increase in sPD-1 level during erlotinib therapy had statistically significant longer PFS and OS, compared to patients with decreasing or undetectable sPD-1 [35]. They did not find this correlation between sPD-1 levels and the emergence of a T790M mutation [35].

Another study showed improved PFS and OS for NSCLC patients with a stable level of sPD-1 or an increase in sPD-1 after treatment with two cycles of nivolumab [36]. The underlying mechanism for increased levels of sPD-1 after certain modalities of treatment (radiotherapy, anti-EGFR therapy, and anti-PD-1 immunotherapy) may be the reactivation of tumor-specific cytotoxic T-lymphocytes [36]. These cells make up the primary source of sPD-1 in circulation [36]. Reactivation can result at least partly from increased antigen presentation by APCs, upregulation of HLA-I on tumor cells, and alleviating T-cells’ inhibition after treatments such as radiotherapy, EGFR TKIs, and anti-PD-1 immunotherapy [33]. A similar mechanism of action could be at the basis of the action of sPD-L1.

A comparative view of different studies with cut-off values used for sPD-L1 is presented in Table 3.

The limitations of our study are the small number of patients and the different reference values for sPD-L1 compared to other studies. We included 35 patients who achieved disease control, because they were alive at the moment the analysis was performed. Our study bring inconsistent data relative to sPD-L1 as a predictive biomarker, at first in patients treated with chemotherapy but now also in patients treated with TKIs and immunotherapies. The major limitation of sPD-L1 as a biomarker in the context of clinics, besides the inconsistent data, is the absence of standardization of sPD-L1 measurement, as several ELISA kits and different thresholds were used in the studys. Thus, it is impossible to make a comparison of the results and to make a conclusion about the reference range. It is necessary to perform another study with a larger number of patients. Furthermore, it would be interesting to see the impact on the results if the samples were also collected at baseline and if patients who did not have a response to treatment were included in the analysis.

## 5. Conclusions

In our study, we found that patients with high sPD-L1 had numerically better PFS and OS, but there was no statistical significance; however, this is not in concordance with the results of the majority of previous studies.

Further studies with a larger study population are needed for better understanding the potential role of sPD-L1 as a predictive non-invasive biomarker in EGFRmut NSCLC.

## Figures and Tables

**Table 1:** Comparative view of demographic characteristics for groups of patients with low and high sPD-L1.

Demographic Characteristics	sPD-L1 ≤ 182.5 ng/L *n* = 25	sPD-L1 ˃ 182.5 ng/L *n* = 10	* p * -Value
Age, mean ± sd	63.5	63.4	0.976
Female sex, n (%)	17 (68%)	7 (70%)	
Male sex, n (%)	8 (32%)	3 (30%)	
Smoking, n (%)			0.354
Never smokers	14 (56%)	5 (50%)	
Former smokers	6 (24%)	3 (30%)	
Current smokers	5 (20%)	2 (20%)	
EGFR mutation			0.749
Del 19	13 (52%)	7 (70%)	
L858R point mutation exon 21	9 (36%)	2 (20%)	
Rare mutations	3 (12%)	1 (10%)	
Del 20	1		
G719X exon 18	1	1	
G719X exon 18 and S768I exon 20	1		

**Table 2:** Response to EGFR TKIs by group (*p* = 0.206).

Variable	sPD-L1 ≤ 182.5 ng/L N = 25	sPD-L1 ˃ 182.5 ng/L N = 10
Complete response	0/25	1/10 (10%)
Partial response	12/25 (48%)	2/10 (20%)
Response	12/25 (48%)	3/10 (30%)
Stable disease	13/25 (52%)	7/10 (70%)
Disease control	25/25 (100%)	10/10 (100%)

**Table 3:** Comparative view of different studies and cut-off values used for sPD-L1.

Study	Patient Population	sPD-L1 Cut-Off Value	Findings
Ceriman Krstic et al.	EGFRmut NSCLC patients who achieved disease control	182.5 ng/L	Pts with higher levels had better outcomes, but there was no statistical significance.
Jin et al. [22]	SCLC	7.0 ng/mL	Pts with higher levels had no response to CT.
Okuma et al. [2]	Advanced lung cancer	7.32 ng/mL	Pts with high levels had worse prognoses.
Jia et al. [1]	EGFRmut lung adenocarcinoma	568.19 pg/mL	Pts with high baseline sPD-L1 levels and patients with high on-treatment sPD-L1 levels had shortened PFS, but there was no statistical significance.
Zhang et al. [17]	NSCLC, healthy controls	0.636 ng/mL	Pts with high levels had worse prognoses; the same results were observed for the EGFRmut group, but there was no statistical significance.
Zhao et al. [25]	Advanced NSCLC	0.0965 ng/mL	Lower levels of baseline sPD-L1 correlated with longer OS; sPD-L1 levels were significantly lower at week 2 and week 4 compared to baseline, but afterward, RT levels were similar to baseline.
Okuma et al. [26]	NSCLC	3.357 ng/mL	Pts with high levels had worse prognoses.
Costantini et al. [27]	Advanced NSCLC (EGFRwt, ALK negat)	0.0337 ng/mL	Pts with high levels had worse prognoses.
Tiako Meyo et al. [32]	EGFRwt vs. EGFRmut	0.156 ng/mL	Positive levels of sPD-L1 in pts treated with nivolumab correlated with shorter PFS. Such correlation was not found in the EGFRmut group of patients. After two cycles of nivolumab, pts with increased or stable levels of sPD-1 had longer PFS and OS.
Zheng et al. [33]	Advanced gastric cancer	0.5993 ng/mL	Pts with higher levels of sPD-L1 had better prognoses.
Vecchiarelli et al. [34]	Advanced NSCLC, healthy controls	37.81 pg/mL	Pts treated with CT had significant increases in median sPD-L1 after 3 months of treatment. Pts treated with TKIs and immunotherapy did not have significant changes in their median sPD-L1 levels. No significant differences in ORR, PFS, and OS were found between patients with high and low sPD-L1 levels.

## Data Availability

The data presented in this study are available upon request from the corresponding author (who will accurately indicate the status of the data).
